# PDFDataExtractor: A Tool for Reading Scientific Text and Interpreting Metadata from the Typeset Literature in the Portable Document Format

**DOI:** 10.1021/acs.jcim.1c01198

**Published:** 2022-03-29

**Authors:** Miao Zhu, Jacqueline M. Cole

**Affiliations:** † Cavendish Laboratory, Department of Physics, University of Cambridge, J. J. Thomson Avenue, Cambridge CB3 0HE, U.K.; ‡ ISIS Neutron and Muon Source, STFC Rutherford Appleton Laboratory, Harwell Science and Innovation Campus, Didcot, Oxfordshire OX11 0QX, U.K.; § Department of Chemical Engineering and Biotechnology, University of Cambridge, West Cambridge Site, Philippa Fawcett Drive, Cambridge CB3 0AS, U.K.

## Abstract

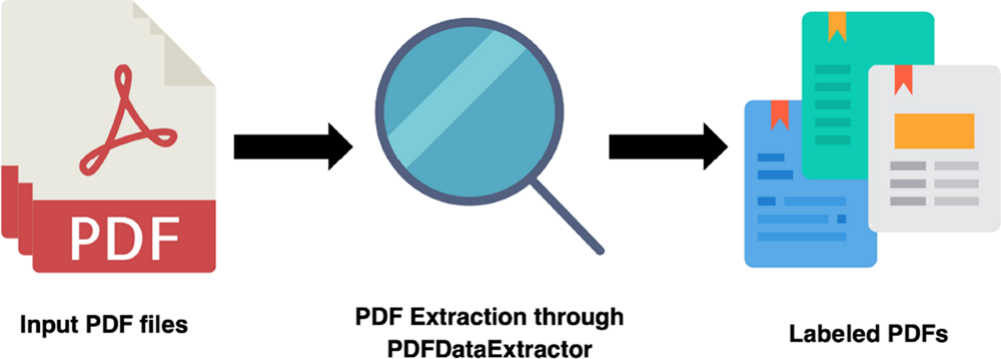

The layout of portable document format (PDF) files is constant to any screen, and the metadata therein are latent, compared to mark-up languages such as HTML and XML. No semantic tags are usually provided, and a PDF file is not designed to be edited or its data interpreted by software. However, data held in PDF files need to be extracted in order to comply with open-source data requirements that are now government-regulated. In the chemical domain, related chemical and property data also need to be found, and their correlations need to be exploited to enable data science in areas such as data-driven materials discovery. Such relationships may be realized using text-mining software such as the “chemistry-aware” natural-language-processing tool, ChemDataExtractor; however, this tool has limited data-extraction capabilities from PDF files. This study presents the PDFDataExtractor tool, which can act as a plug-in to ChemDataExtractor. It outperforms other PDF-extraction tools for the chemical literature by coupling its functionalities to the chemical-named entity-recognition capabilities of ChemDataExtractor. The intrinsic PDF-reading abilities of ChemDataExtractor are much improved. The system features a template-based architecture. This enables semantic information to be extracted from the PDF files of scientific articles in order to reconstruct the logical structure of articles. While other existing PDF-extracting tools focus on quantity mining, this template-based system is more focused on quality mining on different layouts. PDFDataExtractor outputs information in JSON and plain text, including the metadata of a PDF file, such as paper title, authors, affiliation, email, abstract, keywords, journal, year, document object identifier (DOI), reference, and issue number. With a self-created evaluation article set, PDFDataExtractor achieved promising precision for all key assessed metadata areas of the document text.

## Introduction

The number of publications has increasingly grown since the digitalization of publishing,^1^ providing a more efficient platform for scientific communities to share research results. This large number of publications has led to the literature becoming a form of “Big Data.” Such data are useful to data science, which has evolved into a research field owing to the stepwise increase in data capacity for high-performance computing, and the increasing availability of open-source scientific data and software code. It is exciting to realize that the field of “Big Data” has emerged to produce exciting opportunities for discovering new science from patterns found in large arrays of data. Such patterns are best found when data are mined from a structured assembly of information (a database) that contains the most relevant information about the problem in hand.

However, related data are difficult to collate. This is because researchers typically share scientific results through many distinct reports, which can take a variety of forms such as academic papers, technical reports, books, patents, dissertations, or theses. Data are thus strewn across scientific documents in a highly fragmented form. A document may feature unstructured data (e.g., in-line text) or semistructured data (e.g., a table of information), while related data may span many documents. Related data need to be structured and collated in a fashion that auto-builds a database in order to become useful. Text-mining tools that employ natural-language processing (NLP) have enabled the structuring and collation of related data. Open-source software packages, such as CoreNLP^2^ and Spacy,^3^ can mine text that uses general language. However, such tools perform poorly when applied to the scientific domain, owing to its highly specialized language and writing style. The “chemistry-aware” NLP-based text-mining tool, ChemDataExtractor,^4^ was created to overcome this limitation.

ChemDataExtractor^4^ uses an NLP-enabled workflow that is geared specifically to mine chemistry-related information from publications. ChemDataExtractor^4^ performs best in the scientific literature that is imported as mark-up language, for example, HTML or XML. This is because the literature provided in the HTML or XML format is suitable for parsing in sections that are semantically marked.^5^ For example, *“PDFDataExtractor: A Tool for Reading Scientific Text and Interpreting Metadata from the Typeset Literature in the Portable Document Format”* in this document would be tagged as “*title*” in mark-up language. Other sections like headings, paragraphs, captions, and tables are also tagged in the literature. Therefore, once combined with auxiliary semantic information, it is possible to perform analysis on one or more user-defined specific sections. Thus, textual noise from document features such as headers, page numbers, and author affiliations can be prevented from being fed into the extraction pipeline.

Unlike HTML and XML, the layout of the literature provided in the portable document format (PDF) stays the same across all different viewing devices.^5^ No semantic tags are usually provided in PDF files^6^ as the text within this format was not originally designed to be read or interpreted by software programs. Nevertheless, many NLP applications rely on semantic information of text fed into the pipeline.^7^ For example, it is essential to correctly identify the semantic roles of text blocks from the literature if one solely wants to perform NLP analysis on abstracts or to find affiliations of authors from references of a given number of scientific documents that are provided in the PDF; that way, only text from the abstract or reference sections of each document are fed to a software program for extraction and analysis.^8^ Although most articles can be accessed through HTML or XML, there are a large number of articles that can only be accessed in PDF^5^. Services such as literature mining and database creation rely on accurate metadata from articles. However, metadata are sometimes missing.^9^

ChemDataExtractor^4^ only has very limited proficiency for extracting and interpreting data from PDF files. This limited functionality relies on a PDF-layout-analysis tool called PDFMiner^10^ to process input files. PDFMiner^10^ is a PDF-file extraction tool that outputs excellent results in terms of PDF-layout analysis, that is, representing a PDF file in many text blocks with correct reading sequences. It spaces the positions and fonts of the individual characters.^6^ However, the extraction ability of PDFMiner^10^ is primitive, where limited semantic information about text blocks is extracted. This is because PDFMiner^10^ is essentially a structural-analysis package, and no identification of the logical role of text blocks is performed.^11^

Extracting information from PDF files is well studied, and notable results have been achieved from previous studies. Available data-extraction solutions usually tackle problems by utilizing either rule-based or machine-learning-based approaches. PDFMiner,^10^ PDFX,^1^ pdftotext,^12^ and PDFExtract^13^ represent the rule-based approaches to convert PDF files. These tools generally use a combination of visual and text/content information to reconstruct the logical representation of a PDF file. Machine learning has increasingly drawn more attention in almost every research field. For example, Cermine,^9^ ParsCit,^14^ and GROBID^15^ are excellent tools for information retrieval, which use techniques like support vector machines (SVMs) and conditional random fields (CRFs) to classify text. Overall, these solutions tend to use generalized methods to cover different layouts. They offer fair extraction results with different emphases. However, the driving force behind the creation of PDFDataExtractor presented herein is to (1) create and populate databases and (2) serve as a new PDF extraction plug-in in ChemDataExtractor,^4^ which hosts metadata rather than simply perform PDF layout analysis. To this end, we present PDFDataExtractor, a tool that extracts metadata from scientific articles using PDFMiner^10^ to build text blocks.

This study shows how this is possible via a template-based approach. Templates can push PDF extraction limits further at the expense of handling more layouts. When building a database, precision is considered to be a more important factor than recall, affording a template-based approach suitable. The modular system of PDFDataExtractor also facilitates future customization. Overall, five large publishers account for more than 70% of chemistry and nearly 40% of physics fields of research.^16^

**Figure 1:**
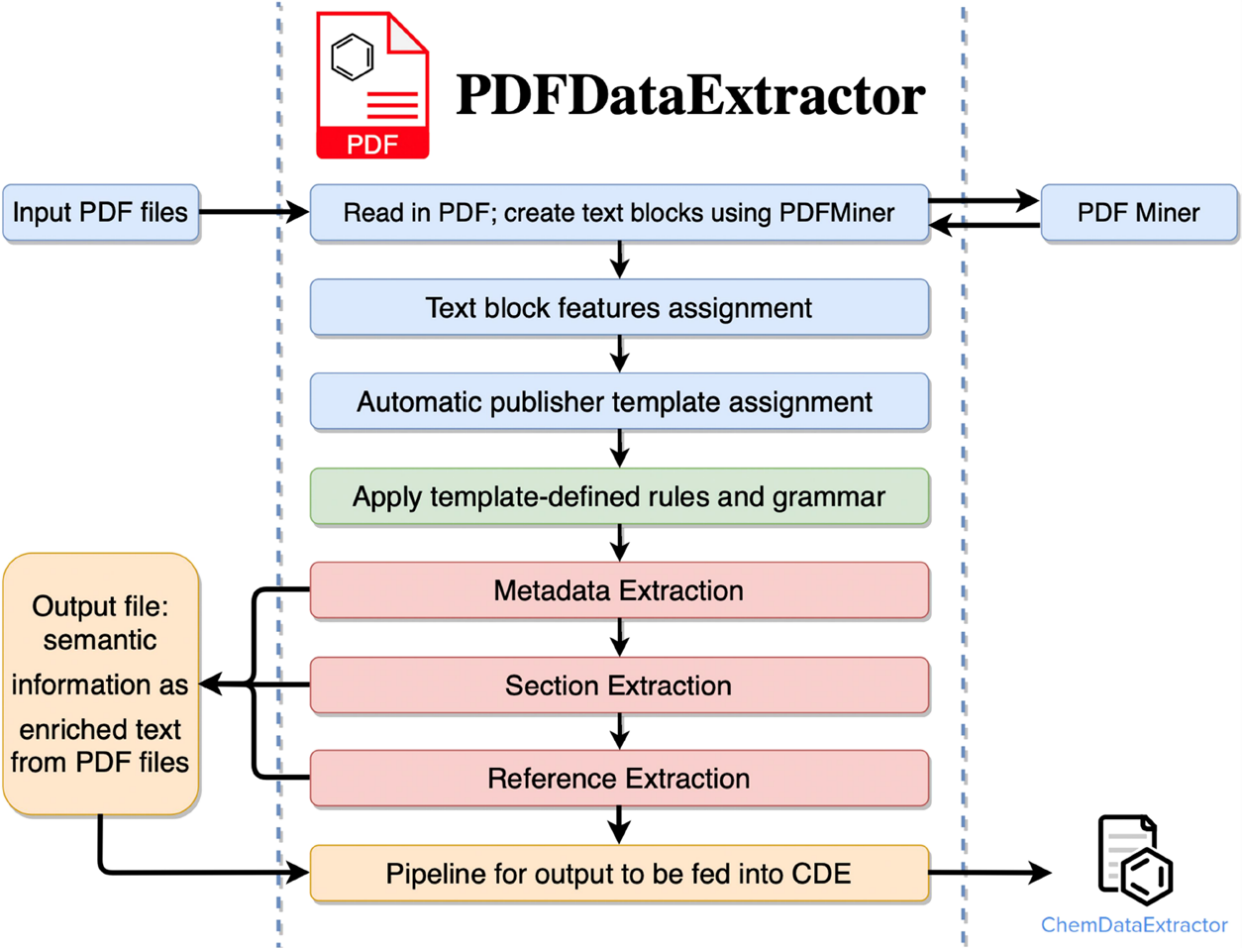
High-level schematic workflow of PDFDataExtractor.

PDFDataExtractor has been created as a plug-in to ChemDataExtractor.^4^ This allows PDF-file data extraction to be channeled directly into a text-mining pipeline with chemical-named entity-recognition capabilities, as realized by ChemDataExtractor.^4^ PDFDataExtractor also generates metadata that are useful for various ChemDataExtractor^4^ functions that require knowledge of the sectioning of a document. PDFDataExtractor can generate metadata for 13 primary logical parts that can principally represent a scientific publication: these are the title, author, abstract, section headings, paragraph of the body text, figure, header, caption, journal information, DOI, page number, acknowledgments, and references (Figure 1).

## System Overview

**Figure 2:**
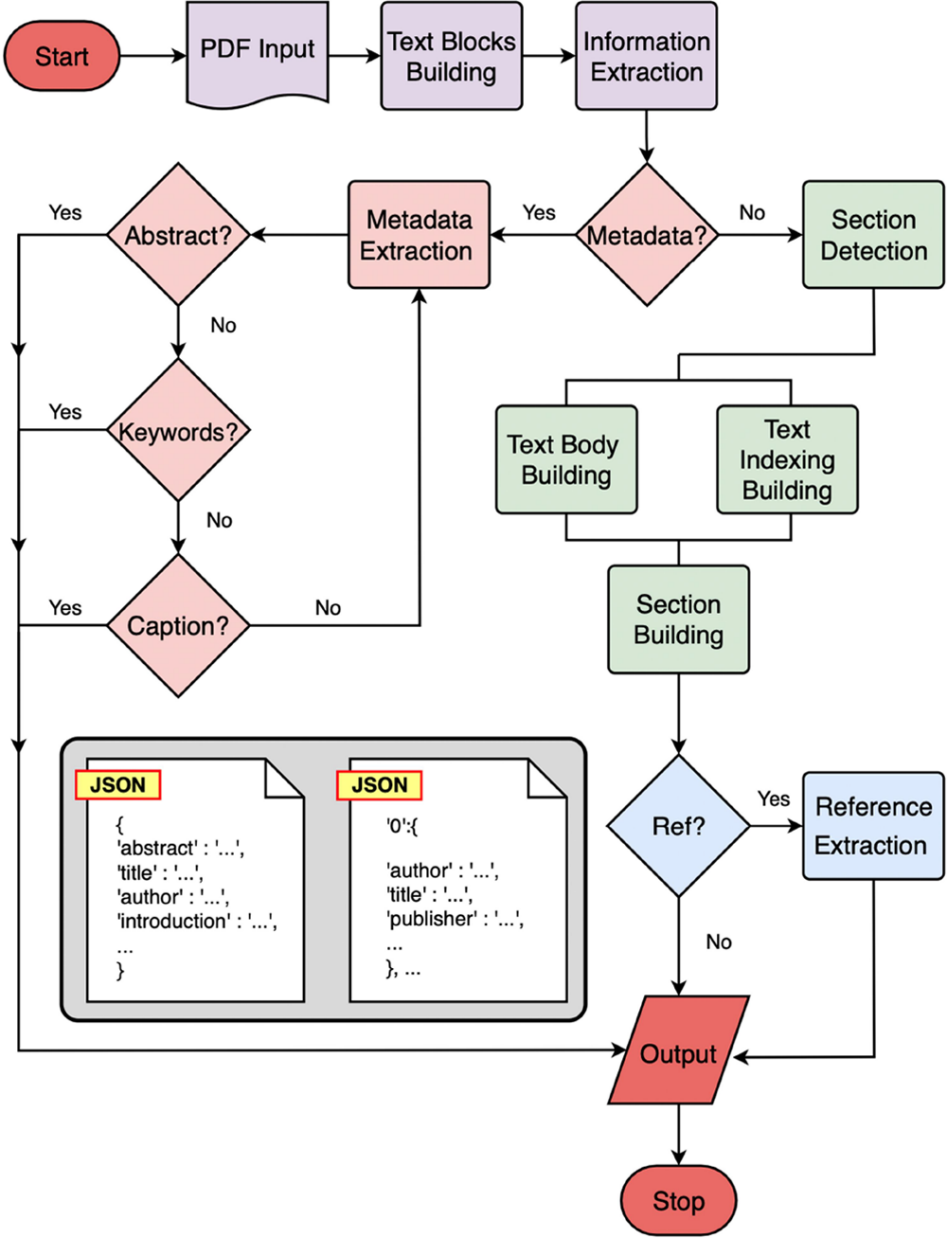
Low-level schematic workflow of PDFDataExtractor.

**Figure 3:**
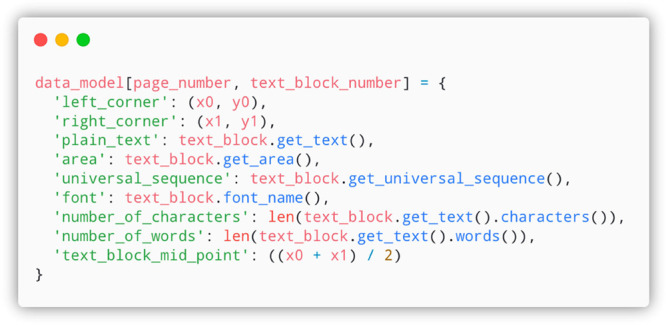
Schematic pseudocode data model showing features of a preprocessed text block.

The general workflow consists of five principal stages: (1) the preprocessing of text; (2) metadata extraction; (3) documentation section detection; (4) reference detection; and (5) output extracted results to ChemDataExtractor.^4^ The extraction process begins with a PDF being fed to PDFMiner,^10^ where it is converted into a representation of text blocks in the correct reading order. Each text block is assigned a universal numbering label in PDFDataExtractor, which is later used to segment the text body. Thus, for each text block, more features are generated by PDFDataExtractor, based on the information provided by PDFMiner,^10^ as shown in Figures 2 and 3, which is essential for all its later extraction stages. Then, PDFDataExtractor automatically picks a predefined extraction template and passes it to the subsequent extraction components. Following this preprocessing stage, the program then loops through text blocks on the first page of the document in order to determine if they include metadata. If this is the case, the text block is fed to the metadata-extraction stage of the pipeline, and the extracted information is attached sequentially to the output result. In the case that the text block does not contain metadata, a separate section detection is performed to extract the body section titles, which is accomplished by simultaneously constructing the text body and indexing it. Afterward, the extracted reference text is fed to the reference extraction component for citation parsing. Finally, extracted results are fed into ChemDataExtractor^4^ for chemical information extraction.

### Preprocessing of Text

**Figure 4:**
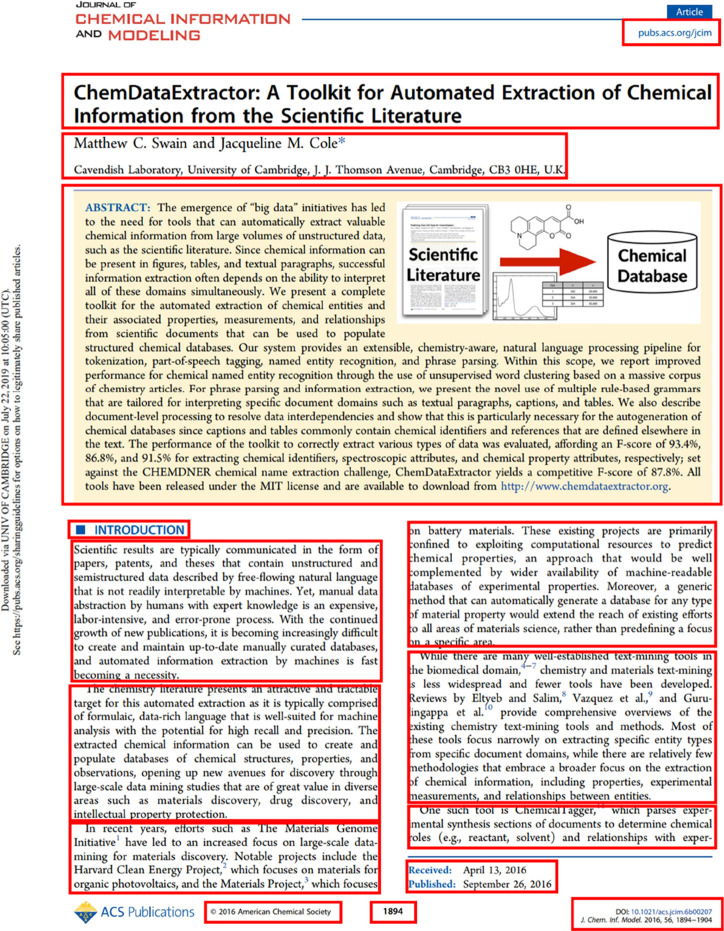
Schematic presentation of text blocks extracted by PDFMiner.^8^

It is necessary to preprocess text blocks and present the entire PDF in a correct data model (Figure 3) prior to any information extraction. By default, PDFMiner^10^ processes the document page by page. It is like a blank sheet of paper is used every time a page is turned. This means that everything is refreshed, and there is no link between pages; simply, those text blocks are not in sequence at the documentation level. To this end, each text block is assigned a universal sequence number to present its relative location at the document level, providing the ability to index and select text blocks across different pages. One way to understand the universal sequence number is to imagine that all pages are flattened into a single page, where the sequence number of text blocks proceeds sequentially from 0, with an interval of 1. Figure 4 schematically shows distinct types of extracted text blocks by PDFMiner.^10^

Each text block is stored as a key-value pair, as shown in Figure 3. The key is a combination of the current page number being processed and the text block within the page. This enables quick indexing of text blocks across the whole document, in addition to restricting the pages that are processed. The value is a subdictionary containing all the features of one text block that are selectively used by later-processing components to extract information. For example, “number_of_word” is used by the metadata-extraction component to extract the abstract, and “universal_sequence” is used by the section-detection component for indexing the sections. Some text blocks, such as headers and page numbers, contribute no information to the extraction pipeline and are therefore removed at later stages. Notably, not all features of the text blocks are used by the extraction pipeline; indeed, many of these might be useful for future development of the program.

### Metadata Extraction

Extraction of semantic information begins once all text blocks have been preprocessed and a template is selected by PDFDataExtractor. Text blocks are initially fed to the metadata-extraction stage of the pipeline, where the abstract, title, keywords, and caption are extracted using a combination of predefined rules and grammar, which are discussed in the following subsections. Such metadata are usually displayed on the first page of a PDF; thus, part of the extraction process is restricted to this page by indexing the page number of each text block.

#### Abstract Extraction

**Figure 5:**
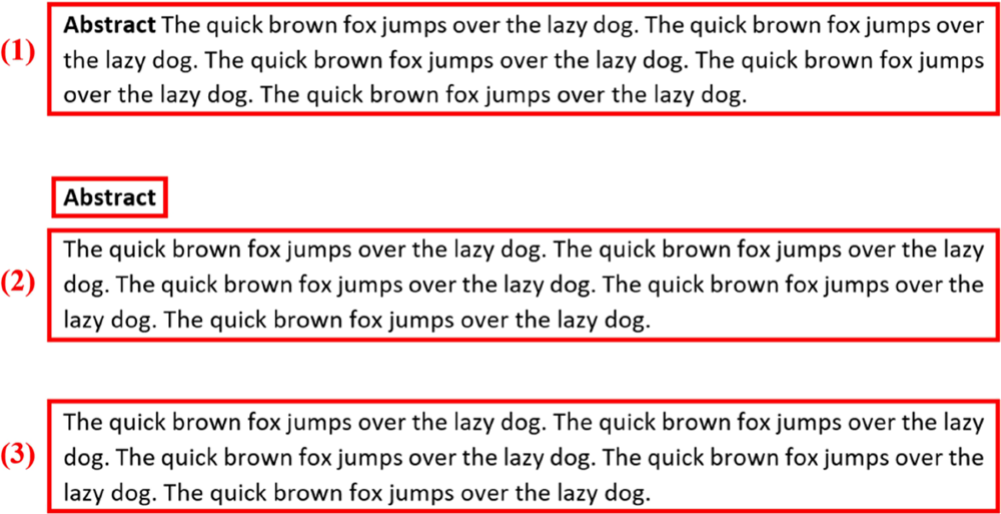
Three cases of usually seen abstracts from scientific articles.

The abstract-extraction component of the program can handle three different situations, which are indicated in Figure 5. This consists of (1) one text block containing the string “abstract” within; (2) two text blocks with string “abstract” separated; and (3) one text block with no string “abstract.”

These three situations represent the majority of abstract stylings and are shown in Figure 5. The preprocessed text blocks from the first page of the PDF are the input source for abstract extraction. The program checks each text block and automatically selects the appropriate method for the extraction of the abstract.

In the first situation, indicated as (1) in Figure 5, the program can locate the target text if one text block contains the string “abstract” and the length of the block satisfies the predefined threshold.

In the second situation, the string “abstract” is separated from the target text, resulting in two text blocks designated as “abstract” and “target text”, shown as (2) in Figure 5. An “identifier” is used in this case, which contains the coordinates of the first “abstract” text block. The program then compares the coordinates of each text block with the “identifier.” The target text is found once the coordinates of one text block are within the allowed error when compared to the “identifier.” In other words, a target text block is identified once the text block vertically aligns with the identifier “abstract” text block. However, errors can occur in this situation because the two text blocks are not always perfectly aligned.

In the third situation, as shown as (3) in Figure 5, where no “abstract” string is included in text blocks, the program works based on the assumption that the abstract will occupy the largest area on the first page with other rules; the combination of such rules varies among different publishers. Therefore, the program compares the area of each text block, with the result being continuously updated until the target text block is found. In this case, it is highly likely to select the “introduction” on the first page. Thus, postextraction filtration is applied before assigning the result to the final output.

#### Title Extraction

**Figure 6:**
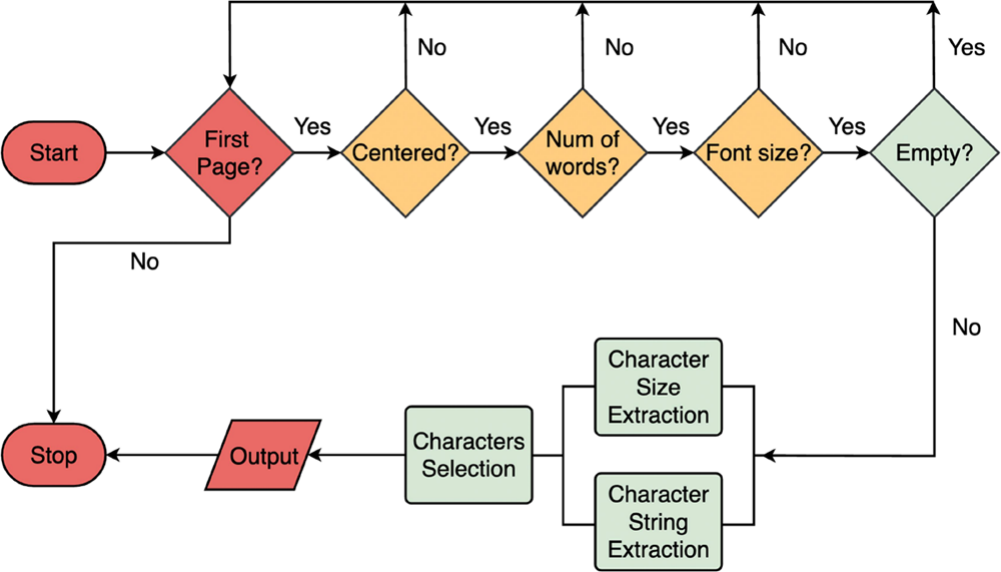
Schematic title-extraction workflow.

The title-extraction workflow (Figure 6) assumes that the target text block is generally expected to be located at the top center of the first page. This pipeline first selects text blocks that are centered on the first page and then filters out text blocks that do not meet the length requirement. Finally, a font size filter is applied. Nonetheless, certain publishers, such as Elsevier, slightly offset their title text. Hence, their article titles are not perfectly centered on the page, and the extracted text block might contain unnecessary data or “noise”, such as the type of the document, author names, and institute affiliations. To this end, an extra step is added. Such an extra step analyzes each character from the extracted text block and performs a parallel character string and size construction. It assigns each character with its own font size, and then, only characters that satisfy the font size requirement are selected.

#### Keywords and Caption Extraction

**Figure 7:**
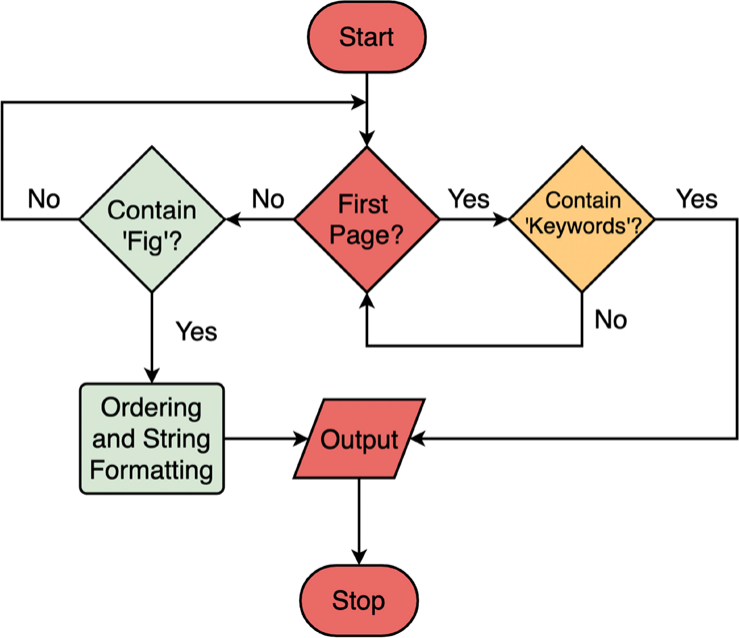
Schematic extraction flow for captions, keywords, and so forth.

The extraction of keywords and captions is more straightforward than other processes. In the case of keywords, the text block usually contains the string “keywords,” while for captions, each text block usually starts with “Figure X” and naturally forms a separate block from the rest of the text. Therefore, the text block for both cases usually contains no noise, and the extraction can be achieved with simple rules and grammar. An extraction flow chart for keywords and captions is shown in Figure 7. Each caption is then assigned a sequence number for sorting.

### Documentation-Section Detection

This stage of the extraction pipeline has two main components that are “noise-search-pattern creation” and “main-body-text segmentation.”

#### Noise-Search-Pattern Creation

**Figure 8:**
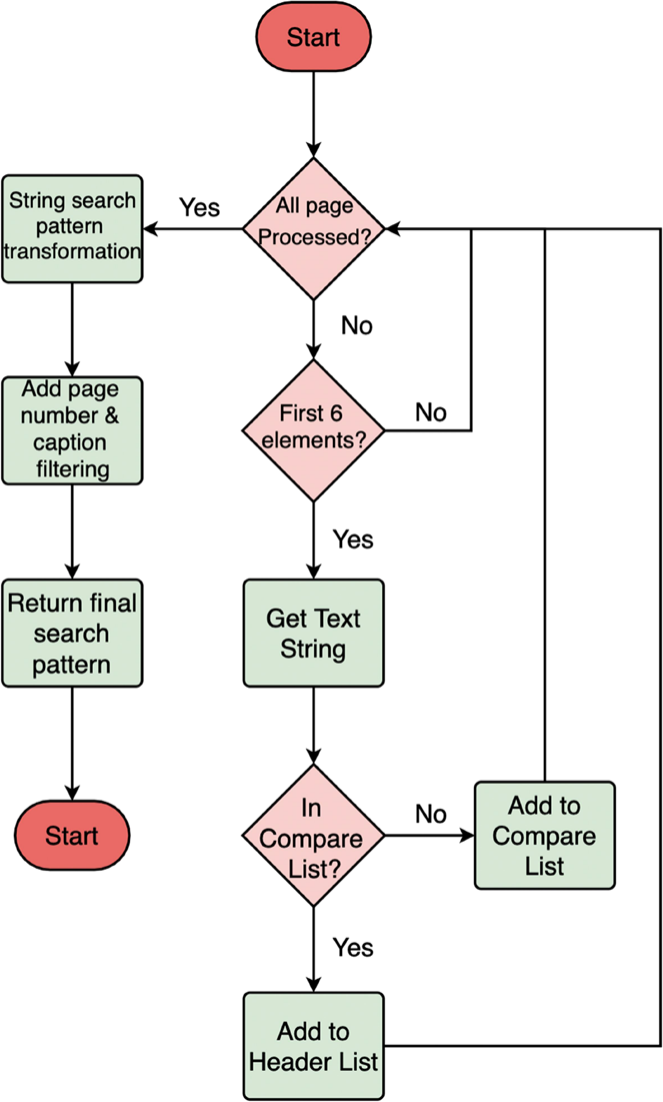
Schematic of the noise-search-pattern-creation workflow.

The noise-search component of the pipeline is used to exclude noisy information such as the header, page number, and so forth; it then converts these into a machine-readable search pattern. Such a pattern is then fed to the main-body-text-segmentation component of the pipeline. The workflow for the noise-search-pattern creation is shown in Figure 8. The program assumes that all headers are located at the beginning of each page. Thus, it takes the first six text blocks from each page into consideration.

A key aspect of the “noise-search-pattern-creation” workflow is the difference between the “compare-list” and “header-list.” The compare-list is used as a buffer to temporarily store candidates, whereas the header-list contains the actual extracted headers. Starting from the first cycle, all of the first six text blocks are appended to the compare-list as a base. Then, for the second and all subsequent cycles, the string of each text block is measured against the compare-list and is then appended to the header-list, provided that matching strings are found. This compare-list is actively updated until the final page is processed. The strings of the extracted headers are automatically converted into machine-readable search patterns once all of the pages have been processed. For example, all of the special characters such as space, period, and parentheses are converted into machine-readable search patterns. Also, captions and page-number search patterns are appended. Finally, this search pattern is ready to be used by the main-body-text-segmentation component of the document-section-detection stage of the extraction pipeline.

#### Main-Body-Text Segmentation

**Figure 9:**
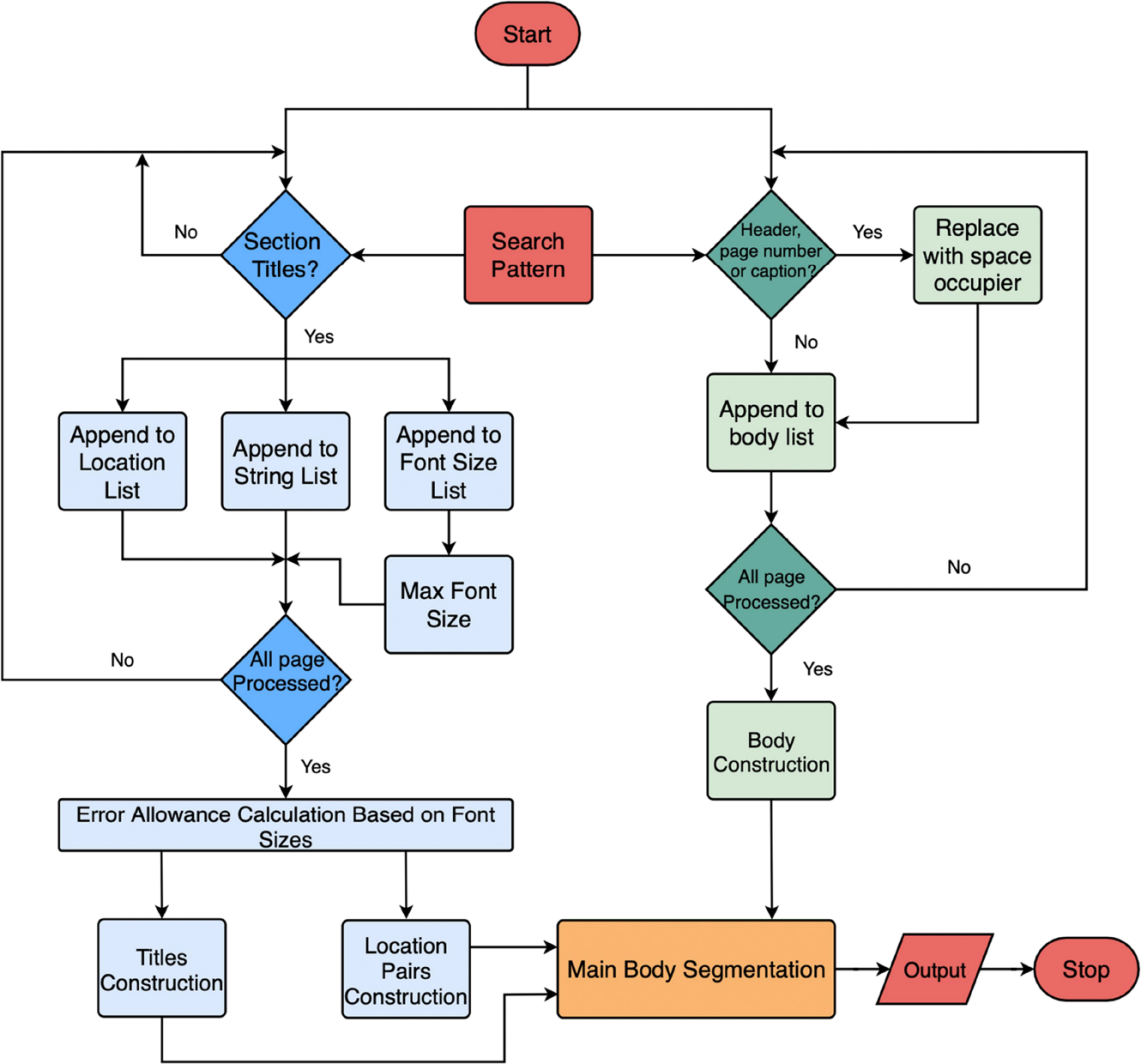
Flow chart showing the main-body-text-segmentation component of the documentation-section detection. Areas colored blue and green represent the location pairs and main-body-text constructions, respectively.

Main-body segmentation takes three items of input: location pairs, titles, and full body text, as shown in Figure 9.

##### Location Pairs and Title Constructions (in Blue)

Location pairs are essentially the location information for each section, which represents the start and end positions of each section. The pipeline first checks each text block and determines if it is a section title by using predefined rules and grammar. Accordingly, the three characteristics of the candidate are appended to three different lists known as “location,” “string,” and “font” lists.

Three lists work accordingly to construct the location pairs. First, the string-list stores the text of candidate text blocks, which is later used for naming the extracted sections. Then, the location-list stores the location information of the extracted text blocks alongside the universal sequence number, which is a document-level feature of one text block and essentially informs the program about the span of one section. Finally, the font-list stores font size information for every candidate text block.

However, the lists must be cleaned before being used by the segmentation component that is indicated in orange in Figure 9. The principle behind the cleaning process is that true positive (TP) candidates have the largest font size among the false positives (FPs).

**Figure 10:**
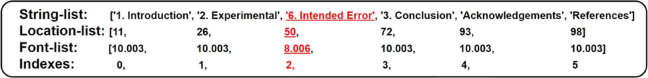
Diagram showing the cleaning process.

Cleaning starts after all pages have been processed. At this point, the three lists are arranged in parallel and are therefore of the same length. At each index, there are three correspondingly linked values, as shown in Figure 10; these are “string,” “location,” and ‘font size’. Cleaning begins with calculating the maximum font size from the font list. Once the maximum font size has been found, the program loops through the font-list to find the indexes of TPs. It is assumed that TPs have the largest font sizes. Once those indexes are extracted, they are then used to filter out FPs in the location and string lists. Consider the example in Figure 10, which contains one intended error marked in red. The function finds that the largest font size is 10.003, and the corresponding indexes are 0, 1, 3, 4, and 5, where 2 is dropped because the font size of this index is smaller than that of all others. Then, string and location are selected based on indexes 0, 1, 3, 4, and 5, where “6. Intended Error” and 50 are dropped.

**Figure 11:**
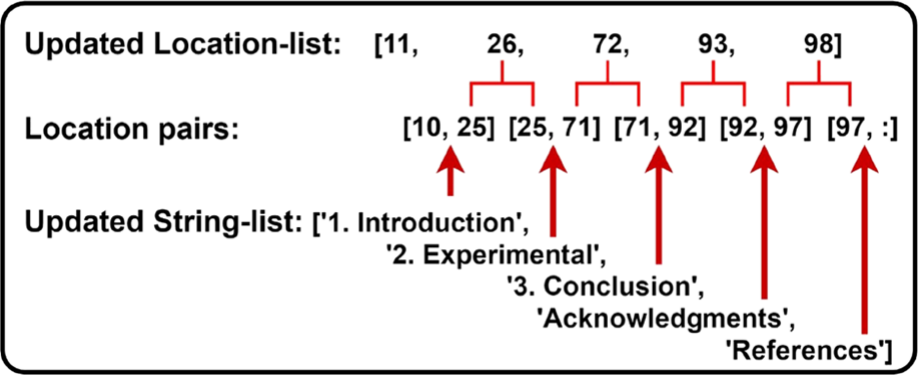
Diagram showing the construction of location pairs that are machine-readable.

Finally, as shown in Figure 11, the updated location-list is then used to construct location pairs that are machine-readable. This is performed by simply inserting each element from location-list into two location pairs. For the first pair, the element is placed at the second place, and for the second pair, the element is placed at the first place. Each location pair informs the program of the start and end positions for each section. For example, “Introduction” is located between the 10th and 25th text blocks. It should be noted that in the first and last identities of the entire sequence of location pairs, “10” and “:” are specifically added to indicate the start and end points of the whole body of the document. In summary, this function returns two lists that are the “location-pair” and the “string” list, which are used by the main-body-segmentation component in the next step. This pipeline conducts the following manipulations:

a) Locating section titles using predefined rules and grammar; storing the corresponding information into location, string, and font lists, which are in parallel.
b) Identifying the maximum font size from the font list and using its index to filter out the FPs in string and location lists.
c) The updated location and string lists are used to build location pairs and name-extracted sections, respectively.

##### Main-Body-Text Construction

Looking at the main-body-text-construction component in Figure 9, as indicated in green, the pipeline first takes the search pattern returned from the noise-creation function and uses it to check every text block to filter out the page number, headers, and captions. If the search is positive, the program removes the corresponding text, and a space occupier is inserted into the body list, as the total number of universal sequence numbers must remain unchanged to match the location pair list. If the search is negative, the string of the text block is appended to the body list until all pages have been processed. The final returned object is a list containing noise-free text from the PDF.

##### Main-Body Segmentation

The main-body-segmentation component of Figure 9, as shown in orange, consists of three lists, two from the location-pair-construction component, namely, string (title) and location-pair, and the full-body-text list from the main-body-text construction. This pipeline segments the full-body-text list using location pairs, as shown in Figure 11. For example, the “Introduction” section spans from the 10th element to the 25th element, meaning that every element within this range is part of the introduction; the same concept applies to the other sections. The title list is then used to name the extracted sections. The essence of the segmentation function is the parallel processing of each text block, whereby each one is processed through two functions simultaneously, and the outputs of the two subfunctions are used together in the final step.

### Reference Detection

**Figure 12:**
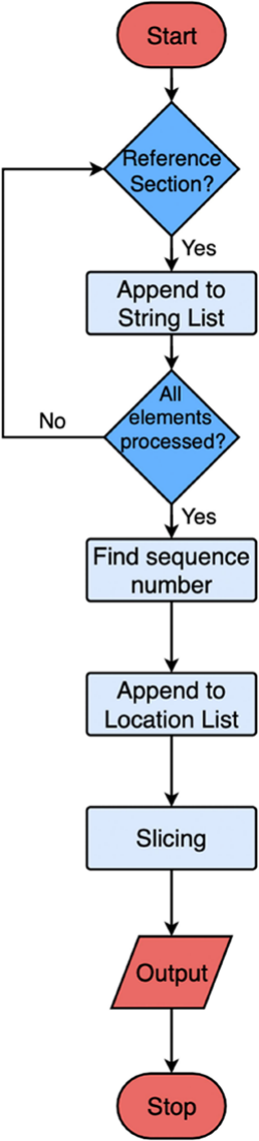
Schematic flow chart for reference extraction.

**Figure 13:**
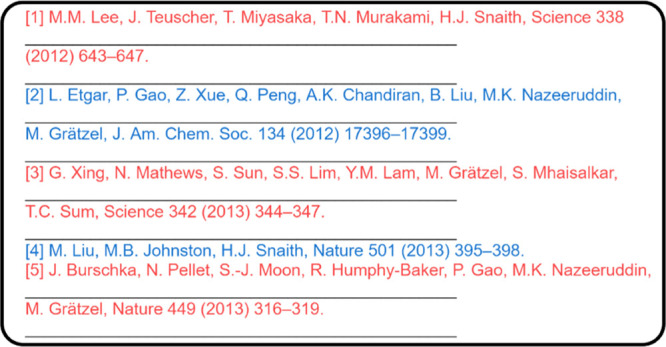
Extracted information is stored as a result list. Alternating colors represent different references, and the black line indicates a different element in the result list.

**Figure 14:**
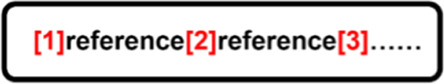
Schematic presentation of flattened citations with the sequence number shown in red.

The reference-extraction workflow is shown in Figure 12. The extracted information is stored as a list that contains all of the text from the reference section but without context between one another, as shown in Figure 13. Entries that are longer than one line can be extracted into two separate elements. Therefore, it is difficult to determine which elements should be grouped together as a single reference entry. To summarize, there is no pattern to follow how each reference will be extracted by PDFMiner,^10^ and one reference entry is very likely to be extracted as more than one part. In this way, the result list can be very complex and fragmented. Therefore, instead of defining many decision functions and rules, the complexity of the information in the results list is lowered by flattening all of the elements into a single long string, as schematically shown in Figure 14.

**Figure 15:**
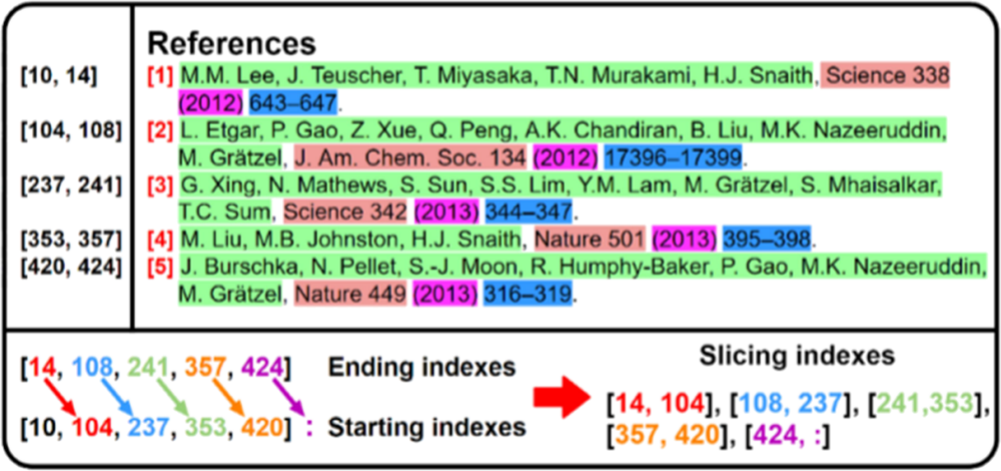
Diagram showing the extraction of metadata from each reference. The upper left section shows the span of the sequence number, and the lower section shows the construction of slicing indexes. The upper right section shows the semantics of each reference entry where authors, journal name, year, and page number are indicated in green, orange, purple, and blue, respectively.

Each reference has already been given a sequence number from the article, as shown in red in Figure 15. Therefore, entries can be extracted by detecting the spans of sequence numbers from the string-list shown in Figure 14.

For example, sequence numbers of references “[1]”, “[2]”, “[3]”, “[4]” and “[5]” have spans of “[10, 14]”, “[104, 108]”, “[237, 241]”, “[353, 357]” and “[420, 424]” and each reference entry can be described with this pattern. Therefore, a single reference entry can be defined as anything that is between two sequence numbers. For machines, this is done by offsetting all of the starting indexes one element away from ending indexes to produce slicing indexes that are used to slice the string-list, as displayed in Figure 15.

### Output Extracted Results to ChemDataExtractor

PDFDataExtractor extracts semantic information from journal articles. However, it can be made “chemistry-aware” to extract chemical entities by feeding extracted information into its parent tool, ChemDataExtractor.^4^

## Evaluation

The performance of PDFDataExtractor was evaluated based on precision, recall, and F-score calculations, as shown below:
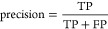
1
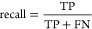
2
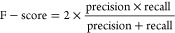
3

TPs are defined as the correctly extracted texts, FPs are defined as the incorrectly extracted texts, and false negatives (FN) are texts that should have been extracted but are not returned by the program. The corresponding XML file is used as the ground truth. Each extracted string is compared against its corresponding ground truth to count as TP if the similarity exceeds a predefined similarity threshold. Such a threshold is set to allow any formatting and textual variations. The similarity is calculated using the Gestalt Pattern Matching algorithm, and eq 4 states the comparison between two strings, where S1 and S2 are the two strings to be compared.
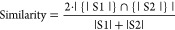
4

We evaluated the performance of PDFDataExtractor against six datasets of journal articles from the American Chemical Society (ACS), Elsevier, the Royal Society of Chemistry (RSC), as well as Angewandte Chemie, Chemistry—A European Journal, and the Advanced Materials family from Wiley. However, creating each dataset to the same size would be impractical because different publishers have different text-mining policies, and some access cannot be gained. Also, the ideal dataset would satisfy the following requirements: (i) It should be easily accessible, which would allow for the creation of large-scale databases; (ii) it should be publicly available to allow every user to perform extraction; (iii) good APIs are necessary when performing large-scale extraction to enhance the coding production efficiency; (iv) it should be large enough and sufficiently diverse to cover various research fields; and (v) it should have good uniformity across different journals that can enhance the extraction performance. Hence, we selected Elsevier as our main evaluation dataset, which covers 10 different research fields with a total of 5797 articles, using search keywords: “solar,” “super alloy,” “Neel temperatures,” “catalysis,” “nano,” “cells,” “light,” “dssc,” “battery,” and “city.” The downloaded Elsevier dataset contains conference papers that are not targeted by PDFDataExtractor at the moment. Therefore, such papers are removed from the extraction.

Meanwhile, the rest of the dataset contains two articles each, with a total of 100 articles.

Image-based articles were filtered out when creating datasets. Datasets store each article in two different formats, one as PDF for extraction, another one as XML or HTML to use as ground-truth data for evaluation against extracted data.

## Results: The Elsevier Dataset

The evaluation of the performance of PDFDataExtractor yields promising results, as shown in Table 1.

**Table 1:** Evaluation Results for Elsevier^a^

dataset	metadata						body		references
Elsevier	title	abstract	doi	journal	keywords	author	sections	captions	refs
precision	77.9	68.9	99.2	60.0	71.3	90.6	57.0	46.0	48.7

a Each cell is the calculated precision.

### Metadata

Overall, the extraction results are promising with each assessed extraction exceeding nearly 60% precision, apart from journal information. The precision for the journal is lower when compared to other types of metadata information. There are several reasons for this deficit in journal information. For example, some authors use slightly different styles such as moving journal information leftwards; in some such cases, this might be visually the same to human eyes but would be different to machines, resulting in it being ignored by the program. Another reason is that some articles downloaded from Elsevier show a completely different layout to the typical format of this publisher. This is because that Elsevier publishes a small number of articles on behalf of another publisher; this also lowers the precision of such articles for abstract extraction. Although each template in PDFDataExtractor is designed to have some generic extraction abilities, handling a completely different layout using the same template is impossible. Sometimes, authors swap the locations of year and volume, which might confuse the program during the evaluation process as the evaluation compares journal name, year, volume, and page fields independently.

For the body and reference, the precision is significantly lower. Such lowering is caused by the ground truth during the evaluation process. For XML files, the labels that mark the corresponding information occasionally target other text, resulting in noisy text being treated as ground truth. Such lowering is eliminated during manual evaluation and can be seen from other datasets.

### Body

PDFDataExtractor can extract article sections and captions with good precision, as shown in Table 1. The text under each section is ignored for evaluation. This is purely because the matching for extracted and ground-truth data can be interrupted by text from images, tables, formulae, and so forth, or simply an encoding of characters. Also, the extraction of metadata information is the main focus of the current version of PDFDataExtractor. When extracting captions, there are two main issues that lower the precision. The first is that the current version of PDFDataExtractor is not able to efficiently separate subcaptions from the main caption. For example, there are captions named Figure 1a,b, and so forth, while PDFDataExtractor treats every subcaption as a whole. The second issue is that PDFDataExtractor includes the string “figure x” in the results, where “x” is the sequence number of each caption. Such noise also contributes to a lowering of the precision.

### Reference

For each reference entry, extracted metadata were discarded. Only plain substrings were used for evaluation against the ground-truth dataset. The precision is lower than the other results. There are several reasons for this, the main one being the noise in ground-truth data, which is impractical to remove. Another reason is that the reading order of each reference entry can be extracted incorrectly, resulting in a comparison of two completely different reference entries during the evaluation stage. Also, PDFDataExtractor is not able to extract reference entries without a sequencing number at the beginning, in a robust manner; the current rules/grammar have their weights more on reference entries with sequencing numbers.

## Results: The Other Datasets

The title, abstract, and DOI extractions for each publisher yield good results with at least 75% precision, except for the RSC (Table 2). This might be caused by the lack of actual “abstract” text; thus, the program struggles to locate the target text block. For email and keywords, some publishers simply do not include such information, and Chemistry—A European Journal puts keywords at the very end of the article, which PDFDataExtractor struggles to process. RSC journals and the Chemistry—A European Journal each give 23 and 27% precision for author extraction, respectively. Such a low precision is potentially caused by the narrow structural spacing between the title, author, and abstract. Sometimes, these text blocks can be extracted as one single text block. For the RSC, Angewandte, and Chemistry—A European Journal, the section title extraction precisions are lower than others. This is because these publishers do not include a sequence number or special character in front of each section title such that the program can be confused. For reference extraction, the Advanced Materials family of journals and the Chemistry—A European Journal outperform others with a precision of 75 and 80%, respectively. Such high results are because each reference entry is assigned a sequence number, which the program can use as anchoring points to separate and extract each reference entry. Overall, PDFDataExtractor performs well on all publishers, especially on metadata information extraction.

**Table 2:** Evaluation results for the ACS, the RSC, the Advanced Materials family, Chemistry-A European Journal, and Angewandte Chemie^a^

dataset	metadata						body		reference
publisher	title	abstract	doi	journal	keywords	author	sections	captions	refs
ACS	0.95	0.90	0.75	0.20	0.50	0.65	0.80	0.90	0.25
RSC	0.83	0.78	1.00	0.00	0.00	0.23	0.25	0.90	0.30
Advanced	0.90	0.90	0.90	0.76	0.80	0.76	0.85	0.75	0.75
Chemistry-A	0.80	0.75	1.00	0.00	0.66	0.27	0.50	0.65	0.80
Angewandte	0.90	0.80	1.00	0.26	0.65	0.50	0.30	0.80	0.10

a Each cell displays the calculated precision.

PDFDataExtractor has several known limitations under certain scenarios, which are as follows:

PDFDataExtractor ignores formulae within an article body and purely treats them as plain text; also, lines within formulae are discarded. The extracted information is essentially correct and at the correct position within body text but without structures. This can be solved by first locating each formula using optical character recognition and then extracting using open-source third-party formula conversion tools.
Tables contain plenty of structured text. However, tables are ignored in PDFDataExtractor, although the information contained in tables is essentially extracted in common with the formula issue. Extraction information from tables is a research topic within its own right. For example, a third-party package termed Camelot^17^ claims to resolve the issue.
Author information is sometimes seen in extracted titles. This is because of the misclassified text blocks from PDFMiner,^10^ where the author text block and the title text block are merged together if the two visually appear to be close. Filters are used to separate titles from the results, but more robust ones are needed.
The reference-extraction component of PDFDataExtractor relies on the sequence number of each reference entry. However, such information is sometimes missing.

## Conclusions

PDFDataExtractor is a plug-in in ChemDataExtractor^4^ for reconstructing PDF articles to ultimately create or autopopulate databases. Its high precision-oriented system design ensures that good-quality databases can be achieved. PDFDataExtractor automatically outputs extracted metadata, article bodies, and references in JSON or the plain text format. These results are then fed into its parent ChemDataExtractor^4^ text-mining software package, for which it is a plug-in tool; this enables data extraction from PDF files to be connected directly to the text mining of chemical information. The customizable modular structure of PDFDataExtractor allows for the user to adapt prewritten templates for specific use.

## Supporting Information Available

Documents used for the evaluation of PDFDataExtractor and the corresponding scripts (ZIP)

## Notes

PDFDataExtractor is released under the MIT license and is available to download from https://github.com/cat-lemonade/PDFDataExtractor. A user guide with examples of code and templates for common chemical publishers is available from https://github.com/cat-lemonade/PDFDataExtractor/demo. Document source information for the documents used for the evaluation of PDFDataExtractor is available at https://github.com/cat-lemonade/PDFDataExtractor/SI.
